# Changes in diet from age 10 to 14 years and prospective associations with school lunch choice

**DOI:** 10.1016/j.appet.2017.05.012

**Published:** 2017-09-01

**Authors:** Eleanor M. Winpenny, Kirsten L. Corder, Andy Jones, Gina L. Ambrosini, Martin White, Esther M.F. van Sluijs

**Affiliations:** a MRC Epidemiology Unit & Centre for Diet and Activity Research (CEDAR), University of Cambridge School of Clinical Medicine, Box 285, Institute of Metabolic Science, Cambridge Biomedical Campus, Cambridge CB2 0QQ, UK; b Norwich Medical School, University of East Anglia, Norwich, Norfolk NR4 7TJ, UK; c MRC Human Nutrition Research, Elsie Widdowson Laboratory, 120 Fulbourn Road, Cambridge CB1 9NL, UK; d School of Population Health, University of Western Australia, 35 Stirling Highway, Crawley, Perth, Western Australia 6009, Australia

**Keywords:** Diet, Nutrition, Adolescence, School, School lunch, Policy

## Abstract

**Background:**

There is limited evidence on how diet changes over the transition from primary to secondary school. In this study we investigated changes in diet from age 10 (2007) to age 14 years (2011) and the contribution of school-time consumption and school lunch choice to such changes.

**Methods:**

The 351 participants with dietary data (4 day food record) available at baseline (age 10 years) and follow-up (age 14 years) were included. Multi-level regression models were fitted for absolute or change in food and nutrient intake, cross-classified by primary and secondary school attended as appropriate, with adjustment for covariates and mis-reporting.

**Results:**

From age 10 to age 14 years, children decreased energy intake from sugars (−2.6% energy (%E)) (standard error (SE) 0.44) and from saturated fats (−0.54%E (SE 0.18)), decreased fruit (−3.13 g/MJ (SE 1.04)) and vegetables (−1.55 g/MJ (SE 0.46)) consumption and increased sugar sweetened beverage (SSB) (4.66  g/MJ (SE 1.87)) and fries (1.31  g/MJ (SE 0.39)) consumption. Intake of snack foods, SSBs, and fries, but also fruits and vegetables was higher outside school hours. Prospective change from non-school lunch to school lunch, compared to maintaining non-school lunch consumption, was associated with decreased consumption of savoury snacks (−8.32 g/day (SE 2.03)), increased consumption of fries (12.8 g/day (SE 4.01)) and decreased consumption of fruit (−25.16 g/day (SE 11.02)) during school hours.

**Conclusions:**

Changes in diet from age 10 to age 14 years differed within and outside of school hours. Consumption of a school lunch, compared to lunch obtained elsewhere, was associated with negative as well as positive changes in diet, suggesting that any efforts to encourage school lunch take-up need to be accompanied by further efforts to improve school lunch provision to meet nutritional guidelines.

## 1 Background

Poor diet is a significant contributor to a number of adverse health outcomes, including obesity, diabetes, cardiovascular diseases and certain cancers (Ambrosini et al., 2012, Appannah et al., 2015, Aune et al., 2011, Franz et al., 2002, He et al., 2007). There is strong evidence that many adolescents do not follow a diet consistent with dietary guidelines; for example, data from the UK National Diet and Nutrition Survey (2008–12) reports that only 10% of males and 7% of females aged 11–18 met the “5-a-day” fruit and vegetable recommendation; and saturated fat consumption on average exceeded the recommended dietary reference values among children aged 11–18 years (Public Health England and Food Standards Agency, 2014).

Dietary intakes have been reported to change through adolescence as children gain independence and control over their food choices, with a number of studies suggesting decreases in diet quality over this period (Nelson et al., 2009, Niemeier et al., 2006). However, there is little evidence from the UK on longitudinal changes in diet quality from late childhood to early adolescence. Data from the Avon Longitudinal Study of Parents and Children (ALPSAC) suggests that diet quality may improve in some respects from age 10 to 13 years, with reductions in intake of saturated fat and free sugars as well as non-core foods and beverages, alongside increasing fibre and fruit and vegetable intake (Emmett & Jones, 2015). However, cohort studies from other countries have found decreases or no significant changes in fruit or vegetable intakes across this age range along with increases or no change in sugary drinks and snacks (Gebremariam et al., 2013, Harris et al., 2015). These differences between studies may result from differences in the period over which data collection took place or differences in country context, suggesting a strong influence of environmental and cultural factors on dietary change at this age.

There has been much interest in the role of the school in restricting poor dietary choices and encouraging healthy choices. Children and adolescents spend a considerable amount of their waking time in school and, moreover, schools have been suggested as an existing social structure that can facilitate implementation of dietary interventions (World Health Organization, 2009). A review of studies of the effect of changes to the school food environment concludes that improvements to the school eating environment can have positive effects on diet quality (Driessen, Cameron, Thornton, Lai, & Barnett, 2014), and further studies have suggested that a healthy school nutrition climate and laws requiring provision of fruit and vegetables in school meals can increase fruit and vegetable intake (Cvjetan et al., 2014, Taber et al., 2013). In England, mandatory standards for school lunches were introduced in 2006, with standards for food other than lunch introduced in 2007. These required the schools to provide a healthy balance of food and drink and banned provision of certain foods (e.g. confectionery, crisps, soft drinks) (Adamson et al., 2013). Since the implementation of these school food standards, a number of studies have suggested that schools can contribute to encouraging a healthier diet through provision of school lunches complying with the standards, which have a better nutritional profile than packed lunches brought from home (Evans et al., 2010, Harrison et al., 2011, Pearce et al., 2011, Spence et al., 2014). However, there has been limited assessment of the overall impact of the school on total diet, and in particular few studies have looked at changing diet from childhood into adolescence in the UK, and the potential role of the school in influencing dietary change.

In this study we make use of data on a cohort of children who reported dietary data before and after their transition from primary to secondary school. Data were collected in 2007 and 2011, shortly after the introduction of mandatory food-based standards for schools in England (Adamson et al., 2013). This paper has two main aims: (1) to investigate changes in dietary intake over this period to understand underlying dietary trends from childhood into adolescence, and (2) to examine the contribution of schools to changing dietary intake over this period, within the context of the school food standards in England. To achieve our second aim we: (a) study the contribution of school-time consumption to overall diet by looking at differences in dietary intake within and outside of school hours; and (b) we examine the impact of changes in school lunch consumption on changes in dietary intake.

## 2 Methods

### 2.1 Study overview and participant recruitment

The SPEEDY study (Sport, Physical activity and Eating behaviour: Environmental Determinants in Young people) is a population-based longitudinal cohort study set in the county of Norfolk, UK (van Sluijs et al., 2008). The analyses presented here use data from baseline (mean age 10 years) and 4-year follow-up (mean age 14 years). Details of SPEEDY participant recruitment and study procedures for data collection have been detailed elsewhere (Corder et al., 2015, van Sluijs et al., 2008). At baseline, primary schools in Norfolk were purposively sampled to achieve urban and rural heterogeneity and 92 schools were recruited and participated in the study. All Year 5 children in these schools were invited to participate. A total of 2064 children provided valid parental consent and were measured at mean age 10 years (57% response rate). At the 4-year follow-up, all participants with valid home addresses (n = 1964) were sent an invitation pack. Researchers additionally gave presentations at secondary schools attended by at least five original SPEEDY participants to encourage participation. Children (n = 480, 24% response rate) were re-recruited at the 4-year follow-up, returning consent forms (signed by both parents and participants) to the study office by post. The University of East Anglia local research ethics committee approved the study.

### 2.2 Data collection procedures

Data collection at age 10 years took place during school visits between April and July 2007 and at age 14 years at schools (or at home if more convenient) between April and July 2011. Researchers visited schools (or homes) to take physical measurements, administer self-report questionnaires and hand out 4-day food diaries. Participants returned the diaries to school 1 week later.

### 2.3 Dietary assessment and processing

Dietary intake was assessed at both time points by participant completion of a food diary, with assistance from their parents. Diet diaries have previously been used and validated with children aged 9–10 years (Crawford, Obarzanek, Morrison, & Sabry, 1994) and applied in young adolescents (Prynne et al., 2013). Diaries were requested to be completed across four consecutive days including 2 weekdays and 2 weekend days. Participants recorded all foods and drinks consumed and the estimated portion size (small, medium or large, number of items or using household measures e.g. number of packets, slices or tablespoons) of each item. In addition, for each eating occasion participants recorded when, where, and with whom they were eating/drinking, what else they were doing at the time (age 14 years only) and from where the food/drink was obtained. At age 14 years, for each diary day, participants further recorded whether the day was a school day (yes/no).

For each food or drink recorded, weights of the portions consumed were approximated using published values for children (Crawley, 2002, Davies et al., 2008, Wrieden et al., 2008). Diaries were coded by a team of researchers at Medical Research Council Human Nutrition Research (Cambridge, UK) using the Diet-In Nutrients-Out (DINO) system, a state of the art integrated dietary assessment system, which uses continually updated British food composition data. DINO incorporates a training programme including Standard Operating Procedures for users to follow, as well as a checking schedule for quality assurance purposes (Fitt et al., 2014).

Dietary data were summarized to give mean daily consumption of macronutrients (as a % of energy intake) and food groups of interest at age 10 years and age 14 years, weighted for weekday and weekend days. In addition, to assess the contribution of food consumed at school to total diet, dietary data was split between food/drinks consumed during school hours and outside of school hours. Consumption during school hours was defined as any consumption between 9am and 2.30pm on weekdays that were indicated to be a school day (age 14 years only) and where children indicated that the consumption took place in school (age 10 years and age 14 years if school day information missing). Consumption outside of school hours included any food consumed outside of school hours (9am–2.30pm) on a school day and food consumed on non-school days, including all weekend days.

In order to investigate changes in diet in detail, we focussed on a selection of nutrient and food group outcomes, rather than an overall measure of diet quality. Dietary outcomes of interest were total energy (MJ), percentage energy (%E) from macronutrients, non-starch polysaccharide (NSP, hereafter referred to as fibre) (grams per Megajoule; g/MJ), sodium (mg/MJ) (not including discretionary sodium) and 6 selected food groups: Confectionery, Savoury snacks, Sugar-sweetened beverages (SSB), Fruits, Vegetables and Potato products. Data on energy, macronutrients, fibre and sodium provide important indicators of overall diet quality, while confectionery, savoury snacks, sugar sweetened beverages (SSBs), and fries are examples of non-core items frequently consumed by adolescents in the UK (Toumpakari, Haase, & Johnson, 2016) and of high policy relevance in this population (Department for Education, 2015).

The confectionery group included chocolate-based products, sugar-based products, sorbets and lollies. Savoury snacks included potato-based snacks, cereal-based snacks, vegetable-based snacks, and savoury biscuits and crackers. SSBs included fruit juice drinks, squashes & fruit concentrates and carbonated soft drinks which were not labelled as ‘sugar free’ or ‘low calorie’. Potato products as a group primarily included potato fries (also hash browns, potato cakes, potato croquettes, potato waffles, potato wedges) and is henceforth referred to as ‘Fries’.

Consumption is presented for total intake, school time intake and non-school time intake, each presented as % of total energy intake, nutrient density (g/MJ) or as average daily grams of a food group consumed per MJ, during that time period. For analysis of the effects of changing between different lunch types, consumption of each food group is presented as average daily grams consumed (not g/MJ) since comparison was made between groups over the same time period, and thus adjustment for change in energy intake was less important to consider, allowing us to use a more interpretable outcome.

Dietary under-reporting is common among adolescents and can bias studied associations (Livingstone, Robson, & Wallace, 2004). We quantified dietary misreporting as the ratio of daily energy intake (EI) relative to estimated energy requirements (EER, estimated as total energy expenditure plus energy required for growth) (EI:EER) (Torun, 2005).

### 2.4 School lunch

Information on whether children obtained their lunch from school, or from outside school was collected from additional questions. At age 10 years, children were asked “On schooldays during lunch break do you generally: (a) eat the lunch served in the school canteen, (b) eat a packed lunch brought from home, (c) go home for lunch or (d) don't each lunch?”. At age 14 years, children were asked the same question, but with additional answer options: (e) Eat lunch you buy outside school from a shop, café, or take-away, (f) Eat something from a vending machine, (g) Eat food from somewhere else (please specify)? We dichotomised the answers to compare school lunch (option a) to lunch obtained from elsewhere (referred to as non-school lunch).

### 2.5 Other measurements taken

Individual-level covariates were included which might affect child dietary intake, including sex, age and SES. SES was assessed as parent-reported level of parental education in three categories: General Certificate of Secondary Education (GCSE) or equivalent or lower (typically taken at age 16 years), General Certificate of Education (GCE) Advanced Level (A-level) or equivalent (typically taken at age 18 years), degree level or higher.

Trained research assistants measured participants’ height to the nearest millimetre using portable Leicester height measures. Body weight was measured to the nearest 0.1 kg using non-segmental bio-impedance scales (Tanita, type TBF-300A). Standard operating procedures were followed. Height and weight measures were used to calculate BMI, which was transformed to standardized z-scores using the least mean square method and 1990 British Growth Reference data (Cole, Freeman, & Preece, 1998).

### 2.6 Statistical analyses

All the analyses were performed using STATA version 14. Children were included in the analysis if they had any dietary data collected on at least 3 diary days, including during school time on at least one day, at both age 10 years and age 14 years, together with data on all covariates.

Age at baseline, gender, BMI z-score and parent education were compared between those included and excluded from the analysis, with t-tests and chi-squared tests used to assess for significant differences between groups.

To describe dietary intakes at 10 and 14 years of age, total, within school and outside school consumption of energy (MJ), macronutrients (%E), fibre density (g/MJ), sodium density (mg/MJ) and food groups of interest (g/MJ) were calculated. Consumption outside of school hours includes weekdays while not at school and weekend days. Dietary intakes during school time and non-school time were compared using paired t-tests. Changes in total, within-school and outside of school intake between 10 and 14 years of age were modelled using linear regression, with change in consumption as the outcome variable, adjusting for gender, age, parental education, and dietary misreporting at age 10 years and age 14 years with random intercepts for level-2 primary and secondary school-level clustering. The model was used to estimate margins at the means of covariates. Average ratio of EI:EER at age 10 years was 0·67 (SD 0·16) and 0.55 (SD 0·16) at age 14 years (correlation coefficient 0.41), indicating that, as is frequently observed in adolescents (Livingstone et al., 2004), under-reporting was very common in this cohort, hence EI:EER at both time points were included in the models as continuous variables (van Sluijs et al., 2016). Intraclass correlation coefficients (ICCs) among groups of students attending the same combinations of primary and secondary school were calculated as the sum of the primary school-level and secondary school-level variances, divided by the sum of primary school-level, secondary school-level and student-level residual error variances (University of Bristol, 2008).

To understand the association between reported school lunch uptake and dietary intakes, we first developed cross-sectional regression models. Total energy intake, macronutrient intake as %E, and fibre and sodium densities were modelled using multilevel mixed-effects linear regression, with primary or secondary school as a level-2 random intercept. Because of the low intake of the food groups of interest, and the skewness of the data which could not be resolved through transformation, we dichotomised food group intake into any consumption vs no consumption. Odds of consumption of each food group was modelled using multilevel mixed-effects logistic regression, again with primary or secondary school as a level-2 random intercept. All models additionally controlled for gender, age, parental education and dietary underreporting as covariates.

We subsequently developed a longitudinal model to look at change in dietary intake by change in usual school lunch type. Those changing from non-school lunch to school lunch were compared to those who took non-school lunch at both time points, while those changing from school lunch to non-school lunch were compared to those taking school lunch at both time points. Changes in dietary outcomes were regressed on changing lunch status, adjusted for food/nutrient consumption at baseline, as well as for gender, age, parental education, underreporting at both time points. All models were cross-classified according to level-2 variables of primary school and secondary school allowing random intercepts for each school group and fitted using linear regression (mle).

## 3 Results

### 3.1 Characterisation of the study population

Of the 480 participants re-recruited at the 4-year follow-up, 388 (80.8%) had diet diary data available at both baseline and follow-up. A further thirty-seven individuals were dropped from these analyses due to lack of information on the secondary school attended (n = 9) or insufficient dietary data (less than 3 days including one school day at each wave) (n = 28). Thus, 351 individuals were included in the study, who attended 78 primary schools at age 10 years and 48 secondary schools at age 14 years. Table 1 shows the characteristics of the individuals included in the study. Those included in the analysis (n = 351) did not differ significantly from those excluded (n = 1713) with respect to sex (44% vs 45% male, respectively; P = 0.86) or SES (parental education category: GCSE or equivalent or below 53.4% vs 59.8%, A-levels or equivalent 28.0% vs 24.2%, degree level or higher 18.7% vs 16.1%; P = 0.09). Those included were however marginally younger (baseline age in years: 10.2 vs 10.3; P = 0.049) and, although not statistically significant, less overweight (baseline BMI z-score: 0.29 vs 0.42; P = 0.05). There were no significant differences between those included in this study and those with dietary data available at baseline only according to percentage energy derived from protein (14.1% vs 14.3%; p = 0.11), fat (33.9% vs 34.0%; p = 0.51) or carbohydrate (52.1% vs 51.6%; p = 0.14).

**Table 1:** Characteristics of those included in the study sample, SPEEDY study, Norfolk, UK, 2007 to 2011.

	Mean/% (SD)
Gender (% girls) (n = 351)	55.6
Baseline age (years) (n = 351)	10.2 (0.31)
BMI (age 10 years) (n = 350)	17.9 (3.05)
BMI (age 14 years) (n = 349)	20.8 (3.85)
BMI z-score (age 10 years)	0.29 (1.13)
BMI z-score (age 14 years)	0.36 (1.18)
Parental education (%) (n = 343)	
GCSE or equivalent or below	53.4
A-levels or equivalent	28.0
Degree level or higher	18.7
% taking school lunch, age 10 years (n = 332)	21.7
% taking school lunch, age 14 years 2 (n = 351)	19.7

Note: BMI = body mass index, GCSE = General Certificate of Secondary Education (typically taken at age 16 years), A-level = Advance level exam (typically taken at age 18 years).

### 3.2 Changes in dietary intake from age 10 to age 14 years

Table 2, Table 3 present unadjusted dietary intake at age 10 years and age 14 years and adjusted change in dietary intake between these ages. Intakes of most nutrients changed by only small amounts from age 10 to age 14 years, with the greatest change for %E from sugars which showed a 11% decrease from baseline. Absolute changes in intake of food groups were small but substantial as a proportion of baseline intake; for example fruit consumption decreased by 27% and vegetable consumption by 21% compared with the median intake at baseline.

**Table 2:** Mean consumption of energy, macronutrients and sodium per day and within and outside school hours, SPEEDY study, Norfolk, UK, 2007 to 2011.

n = 351		age 10 years	age 14 years	Adjusted mean change in consumption
		Mean (SD) #	Mean (SD) #	b (SE) a
Total energy (kJ)	Total daily intake	6488.69 (1440.84)	6786.08 (1956.09)	247.06 (64.62)***
	Daily intake within school hours	2449.99 (828.59)	2492.62 (1083.36)	52.86 (77.19)
	Daily intake outside school hours^b^	4850.25 (1286.5)	5146.74 (1631.33)	251.57 (84.6)**
%E from protein	Total daily intake	14.07 (2.43)	14.07 (2.61)	0.04 (0.20)
	Daily intake within school hours	13.20 (3.92)	12.58 (4.64)	−0.53 (0.32)
	Daily intake outside school hours^b^	14.47 (2.77)***	14.65 (3.01)***	0.24 (0.25)
%E from carbohydrates	Total daily intake	52.08 (4.93)	52.53 (5.72)	0.43 (0.35)
	Daily intake within school hours	51.48 (8.24)	51.41 (9.98)	0.21 (0.75)
	Daily intake outside school hours^b^	52.55 (5.47)*	53.08 (6.39)**	0.45 (0.40)
%E from sugars	Total daily intake	23.58 (5.70)	21.10 (7.13)	−2.64 (0.44)***
	Daily intake within school hours	23.43 (9.68)	20.4 (12.51)	−2.97 (0.81)***
	Daily intake outside school hours^b^	23.64 (6.33)	21.38 (7.76)	−2.46 (0.48)***
%E from fat	Total daily intake	33.86 (4.69)	33.32 (5.4)	−0.56 (0.33)
	Daily intake within school hours	35.35 (8.11)	35.95 (9.29)	0.21 (0.65)
	Daily intake outside school hours^b^	32.98 (5.17)***	32.19 (5.93)***	−0.73 (0.37)*
%E from saturated fatty acids	Total daily intake	13.35 (2.62)	12.83 (2.78)	−0.54 (0.18)**
	Daily intake within school hours	13.48 (4.77)	13.49 (5.19)	−0.15 (0.39)
	Daily intake outside school hours^b^	13.13 (2.74)	12.43 (3.05)***	−0.69 (0.19)***
NSPs (g/MJ)	Total daily intake	1.69 (0.42)	1.75 (0.44)	0.07 (0.03)*
	Daily intake within school hours	1.80 (0.80)	1.78 (0.85)	0.00 (0.08)
	Daily intake outside school hours^b^	1.68 (0.44)**	1.77 (0.50)	0.09 (0.03)**
Sodium (mg/MJ)	Total daily intake	310.86 (58.44)	299.95 (64.96)	−9.06 (5.19)
	Daily intake within school hours	323.91 (102.57)	316.87 (115.28)	−7.20 (7.90)
	Daily intake outside school hours^b^	306.89 (66.62)**	293.92 (73.45)***	−10.85 (5.91)

Abbreviations: SD = standard deviation, b = beta coefficient, SE = standard error, kJ = kilojoules, %E = percentage total energy, NSPs = non-starch polysaccharides, MJ = megajoule total energy intake.

#Paired *t*-test to compare school with non-school consumption * p < 0.05, **p < 0.01, ***p < 0.001; comparison not made for energy intake due to differences in time segment covered.

a Adjusted for gender, age, parental education, mis-reporting and level 2 school variables.

b Daily intake outside school hours includes weekdays while not at school and weekend days.

**Table 3:** Median consumption of food groups per day and within and outside school hours, SPEEDY study, Norfolk, UK, 2007 to 2011.

n = 351 Food Group	Consumption per day	age 10 years	age 14 years	Adjusted mean change in consumption
		Median (IQR)#	Median (IQR)#	b (SE) a
Confectionery (g/MJ)	Total daily intake	2.15 (0.57,4.57)	1.18 (0,3.56)	−0.63 (0.25)*
	Daily intake within school hours	0.00 (0.00,0.00)	0.00 (0.00,1.09)	0.49 (0.56)
	Daily intake outside school hours^b^	2.15 (0.00,4.89)**	0.86 (0.00,3.70)	−0.85 (0.32)**
Savoury snacks (g/MJ)	Total daily intake	1.56 (0.00,2.91)	1.72 (0.00,3.09)	0.26 (0.14)
	Daily intake within school hours	0.00 (0.00,6.57)	0.00 (0.00,7.00)	0.44 (0.35)
	Daily intake outside school hours^b^	0.75 (0.00,2.06)***	0.81 (0.00,1.97)***	0.19 (0.14)
SSBs (g/MJ)	Total daily intake	9.02 (0.00,22.58)	11.17 (0.00,32.42)	4.66 (1.87)*
	Daily intake within school hours	0.00 (0.00,0.00)	0.00 (0.00,0.00)	4.19 (2.29)
	Daily intake outside school hours^b^	8.76 (0.00,22.93)***	12.4 (0.00,34.73)***	4.42 (2.13)*
Fruits (g/MJ)	Total daily intake	11.76 (5.52,22.46)	8.35 (0.79,18.03)	−3.13 (1.14)**
	Daily intake within school hours	15.11 (0.00,35.81)	0.00 (0.00,29.81)	−1.83 (2.64)
	Daily intake outside school hours^b^	8.85 (2.18,19.3)***	4.63 (0.00,13.95)***	−2.8 (1.22)*
Vegetables (g/MJ)	Total daily intake	7.21 (3.10,13.32)	6.39 (2.25,11.45)	−1.55 (0.48)**
	Daily intake within school hours	0.00 (0.00,13.04)	0.00 (0.00,2.11)	−5.42 (1.5)***
	Daily intake outside school hours^b^	7.16 (3.33,13.45)	7.08 (2.56,13.14)***	−0.59 (0.57)
Fries (g/MJ)	Total daily intake	3.41 (0.00,6.60)	3.98 (0.00,9.21)	1.31 (0.39)**
	Daily intake within school hours	0.00 (0.00,0.00)	0.00 (0.00,0.00)	−1.73 (1.09)
	Daily intake outside school hours^b^	2.93 (0.00,7.38)	4.76 (0.00,11.32)***	2.19 (0.47)***

Abbreviations: IQR = inter-quartile range, b = beta coefficient, SE = standard error, MJ = megajoule total energy intake.

#Paired *t*-test compares mean consumption (per MJ energy intake) at school with non-school, *p < 0.05, **p < 0.01, ***p < 0.001.

a Adjusted for gender, age, parental education, mis-reporting and with school attended included as a level 2 variable.

b Non school-time consumption includes weekdays while not at school and weekend days.

The higher %E from fats and saturated fats (at age 14 years only) and lower intake of vegetables (at age 14 years only) during school hours suggest that in some respects school diet may be less healthy than diet outside of school. However consumption of fruits was higher within school than outside school, and consumption of snack foods, SSBs and fries (at age 14 years only) were higher outside compared to within school hours. ICCs for the mean change in consumption of nutrients and food groups among groups of students attending the same primary and secondary schools varied from <0.01 to 0.17 for intake within school hours, and from <0.01 to 0.05 for total daily intake.

### 3.3 Cross-sectional associations between school lunch consumption and diet

Table 4 presents the results of the cross-sectional associations between school lunch consumption and intake of macronutrients and food groups. Associations were largely consistent between the two age-groups. When considering consumption during school hours only, we observed some positive associations of school lunch consumption with diet, including a lower %E intake from sugar and lower odds of consumption of snack foods and SSBs (at age 10 years only) and a higher odds of consumption of vegetables (at age 10 years only) during the school day among those consuming school lunch compared to non-school lunch. However, school lunch consumption was also associated with a lower odds of consumption of fruits, and higher odds of consumption of fries during the school day compared to those not consuming school lunch. Associations between lunch type and total mean daily dietary intakes were more limited. Interestingly, consumption of school lunch at age 10 years was associated with a mean daily energy intake that was on average 234 kJ/day lower than those consuming lunch obtained from elsewhere.

**Table 4:** Differences in food and nutrient intake between students reporting school lunch consumption versus other lunch types, SPEEDY study, Norfolk, UK, 2007 to 2011.

Mean daily consumption	Consumption during school hours		Total daily consumption	
	age 10 years (n = 332)	age 14 years (n = 351)	age 10 years (n = 332)	age 14 years (n = 351)
	beta (SE)	beta (SE)	beta (SE)	beta (SE)
Total energy (kJ)	−256.97 (108.41)*	−54.34 (133.96)	−234.15 (117.78)*	−140.81 (124.26)
%E from protein	2.38 (0.54)***	2.51 (0.61)***	0.20 (0.32)	0.56 (0.35)
%E from carbohydrates	−0.36 (1.13)	−2.26 (1.38)	−0.08 (0.69)	−1.02 (0.79)
%E from sugars	−6.02 (1.28)***	−5.52 (1.68)**	−1.42 (0.79)	−1.66 (0.94)
%E from fat	−1.99 (1.10)	−0.52 (1.28)	−0.1 (0.62)	0.43 (0.74)
%E from saturated fatty acids	−0.76 (0.64)	−0.30 (0.71)	−0.20 (0.34)	−0.12 (0.37)
NSPs (g/MJ)	0.33 (0.11)**	−0.13 (0.12)	0.07 (0.06)	0.03 (0.06)
Sodium (mg/MJ)	−16.56 (14.13)	5.74 (15.63)	−8.44 (8.12)	10.78 (8.85)
Odds of consumption	OR (SE)	OR (SE)	OR (SE)	OR (SE)
Confectionary	0.38 (0.17)*	did not converge	0.87 (0.31)	0.64 (0.19)
Savoury snacks	0.27 (0.09)***	0.25 (0.09)***	0.52 (0.16)*	0.47 (0.17)*
SSBs	0.28 (0.15)*	0.64 (0.23)	0.67 (0.20)	1.07 (0.35)
Fruits	0.26 (0.08)***	0.32 (0.10)***	0.75 (0.34)	0.59 (0.18)
Vegetables	2.63 (0.79)**	0.73 (0.23)	5.20 (5.60)	1.31 (0.59)
Fries	5.42 (1.97)***	6.96 (3.55)***	2.02 (0.70)*	0.64 (0.19)

*p < 0.05, **p < 0.01, ***p < 0.001.

Abbreviations: SE = standard error, kJ = kilojoules, %E = percentage total energy intake, MJ = megajoules total energy intake, OR = odds ratio.

Note: beta is the difference in consumption of energy, macronutrients or sodium for those consuming school lunch compared to those consuming lunch obtained elsewhere, adjusted for sex, age, parental education level, dietary misreporting, and school attended. OR is the odds of any consumption of each food group for those consuming school lunch compared to those consuming lunch obtained elsewhere, adjusted for sex, age, parental education level, dietary misreporting, and with school attended included as a level 2 variable.

### 3.4 Prospective associations between school lunch consumption and diet

Table 5 shows the associations between changing from school lunch to non-school lunch (n = 42) (versus maintained school lunch (n = 30)) or changing from non-school lunch to school lunch (n = 35) (vs maintained non-school lunch (n = 225)) with change in diet over the same time period. The results suggest that changing from non-school lunch to school lunch is associated with a decrease in consumption of savoury snacks and fruits and an increase in consumption of fries. Changing from school lunch to non-school lunch was associated with an increased consumption of savoury snacks, fruits and vegetables and a decreased consumption of fries within school hours. The only significant associations found with total daily food consumption was a decrease in consumption of savoury snacks among those changing from non-school to school lunch.

**Table 5:** Change in lunch type and change in dietary intakes between 10 and 14 years of age, SPEEDY study, Norfolk, UK, 2007 to 2011.

	Consumption during school hours		Total daily consumption	
	Move from non-school to school lunch (n = 35) vs maintain non-school lunch (n = 225)	Move from school to non-school lunch (n = 42) vs maintain school lunch (n = 30)	Move from non-school to school lunch (n = 35) vs maintain non-school lunch (n = 225)	Move from school to non-school lunch (n = 42) vs maintain school lunch (n = 30)
	beta (SE)	beta (SE)	beta (SE)	beta (SE)
Total energy (kJ)	−270.85 (181.19)	143.35 (266.74)	−273 (161.28)	313.97 (206.37)
%E from protein	1.94 (0.76)*	−2.18 (1.43)	0.36 (0.44)	−0.39 (0.63)
%E from carbohydrates	−1.14 (1.93)	1.78 (2.38)	−0.53 (0.99)	−0.86 (1.5)
%E from sugars	−4.07 (2.42)	2.78 (2.25)	−2.11 (1.28)	−1.37 (1.57)
%E from fat	−1.23 (1.77)	0.10 (2.36)	0.09 (0.94)	1.28 (1.38)
%E from saturated fatty acids	−0.79 (0.99)	−0.03 (1.31)	−0.47 (0.49)	0.13 (0.68)
NSPs (g/MJ)	−0.12 (0.17)	0.43 (0.15)**	0.05 (0.08)	0.06 (0.1)
Sodium (mg/MJ)	20.88 (20.51)	38.24 (33.24)	11.08 (11.29)	3.92 (18.53)
Confectionery (g)	−0.47 (3.00)	1.86 (3.93)	−2.07 (4.19)	7.85 (4.97)
Savoury snacks (g)	−8.32 (2.03)***	6.07 (2.95)*	−6.21 (2.47)*	8.84 (4.72)
SSBs (g)	−2.31 (17.98)	12.56 (19.72)	−10.82 (36.61)	1.90 (38.8)
Fruits (g)	−25.16 (11.02)*	27.16 (11.33)*	−7.87 (14.98)	11.99 (20.47)
Vegetables (g)	−4.48 (3.18)	15.21 (6.12)*	−4.13 (7.98)	6.04 (12.05)
Fries (g)	12.80 (4.01)**	−14.27 (5.52)*	11.01 (7.58)	−4.17 (10.77)

*p < 0.05, **p < 0.01, ***p < 0.001.

Abbreviations: SE = standard error, kJ = kilojoules, %E = percentage total energy intake, MJ = megajoule total energy intake.

Note: beta is the difference in each outcome between those who have moved between non-school lunch and school lunch and those who have continued consuming the same lunch type, adjusted for sex, age, parental education level, dietary misreporting at both time points, food/nutrient consumption at baseline and with school attended included as a level 2 variable.

## 4 Discussion

### 4.1 Principal findings

Our study showed both positive and negative changes in overall diet from age 10 to age 14 years, including a decrease in %E consumed from sugars and from saturated fats, but also a decrease in fruit and vegetable consumption and an increase in SSB and fries consumption. Comparing intake during school hours with that outside of school hours, a higher %E from fat at both ages and saturated fat at age 14 years was seen within school hours, and, unlike the overall decrease in saturated fat consumption between waves, no decrease in saturated fat consumption over the study period was seen within school hours. Prospective analyses assessing changes between consumption of school lunch and consumption of lunch obtained from elsewhere corroborate cross-sectional findings, showing associations between school lunch consumption and decreased intake of savoury snacks, but increased intake of fries and decreased intake of fruit, compared to consumption of lunch obtained from elsewhere.

### 4.2 Strengths and limitations of the study

The SPEEDY study comprises one of the largest datasets of longitudinal diet diary data within this age group (Emmett and Jones, 2015, Parkinson et al., 2011). Despite this we did see cohort attrition between age 10 and age 14 years, although the differences seen between those included in our analyses and the baseline sample were small and for the most part not statistically significant, suggesting that this attrition will likely have had limited impact on our findings. This cohort was not, however, designed to be regionally or nationally representative, which should be taken into account when interpreting the results. Nevertheless, this prospective study allows us to assess change in diet in this cohort over time. Diet data was recorded using a multi-day diet diary, considered to be one of the more robust methods of collecting dietary data from adolescents (Burrows, Martin, & Collins, 2010), and allowing us to assess total diet as well as to specifically investigate diet consumed within and outside of school hours. We did find that underreporting was common in our sample, as is often observed in adolescents, and evidence suggests that there may be more underreporting of snacks than main meals (Gemming & Mhurchu, 2016) which may have affected our results.

In order to gain a deeper understanding of changing diet, we performed our analyses across 14 distinct diet outcomes. In such a situation there is the possibility that multiple testing may increase the likelihood of false positive findings. While we have not adjusted the significance level of our findings using a correction factor, in our tables we have reported p-values within several ranges, such that it is possible to interpret the strength of the evidence.

### 4.3 Comparison with previous evidence and implications of the findings

#### 4.3.1 Changes in overall dietary intake from age 10 to 14

There was an indication in our findings that children's diets may have improved in some respects from age 10 to age 14 years. Within the expected overall increase in energy intake over this period, we observed a decrease in %E from sugars and a decrease in %E from saturated fats. However, at both time points the median %E from saturated fat remained above the recommended limit (of no more than 11%E) (Department of Health, 1991), suggesting that considerable further improvement is still required. Our findings are in accordance with published data from the ALSPAC study which showed a reduction in %E intake of saturated fat and from free sugars over a similar age range (Emmett & Jones, 2015). However, in our sample we saw a concomitant decrease in fruit and vegetable consumption, contrasting with an increase observed in the ALSPAC cohort (Emmett & Jones, 2015). It is important to note that the changes in dietary intake seen in this study were all relatively small, at a fraction of a portion of a given food group per day, or a small percentage of total energy intake. However, for some food groups such as fruits and vegetables, given the low levels of baseline consumption, the proportional changes are large. Moreover, others have argued that small changes which persist over periods of time may have substantial effects on changes in weight gain over time (Hill, 2009). These ongoing dietary changes form an important baseline from which to understand the potential associations of diet with school-level factors.

#### 4.3.2 Contribution of school-time consumption to overall dietary intake

The data presented here suggest that energy intake within school hours comprises on average less than 30% of overall energy intake, consistent with previous evidence (Prynne et al., 2013). We observed a higher proportion of energy derived from total fat and saturated fat (at age 14 years only) during school hours, compared to outside of school hours, suggesting that food consumed at school is contributing importantly to the excessive saturated fat consumption among adolescents (Public Health England and Food Standards Agency, 2014). Many of the food groups that we assessed had low consumption within school hours compared to outside of school hours. However, fruit intake was higher during school hours than outside school hours. Previous analysis of data from UK children aged 7–10 has also suggested that they are more likely to eat fruit at school than at home, with no differences seen in place of vegetable consumption (Mak et al., 2012).

Despite the limited contribution of school food to overall energy intake, considerable focus has been placed in recent years on improving the nutritional quality of food offered in schools, and national-level school food policies have been implemented to try to improve the quality of school food. At the time of this study mandatory interim food-based standards for school lunches were already in place with food-based standards for food other than lunch being implemented over the course of this study (Adamson et al., 2013). Even though benefits of the UK school food standards in improving school lunch provision and uptake have been reported by the time of data collection for this study (Evans et al., 2010, Harrison et al., 2011, Pearce et al., 2011, Spence et al., 2014), our findings suggest that further work is still needed to improve children's diet within school hours. Importantly, the school food standards only relate to food provided by the school, and it is challenging for schools to control consumption of foods brought in from outside of school.

#### 4.3.3 School lunch consumption and associations with diet

At both ages, our data show that around 20% of all pupils consumed school lunch, with the remainder bringing lunch into school from home or elsewhere. To better understand the effects of school food provision on school-time consumption, we compared those consuming school lunch with those consuming lunch obtained from elsewhere. We found that at both age 10 and age 14 years, those consuming a school lunch had a lower %E from sugars than those consuming lunch obtained elsewhere. However, we did not see any differences between those consuming school lunches and other lunch types for consumption of saturated fat. Indeed, when looking across food groups we saw a much higher odds of consuming fries among those consuming school lunch than other lunch types. These findings agree with other studies of secondary school lunch, showing that compared to those consuming school lunch, those consuming non-school lunch are more likely to eat fruit but also confectionery and snacks, while those consuming school lunch are more likely to consume vegetables and fries (Pearce et al., 2012, Stevens et al., 2013). Importantly, we saw very limited associations with total diet, suggesting that changes in food consumed during school time may have little effect on overall nutrition throughout the day, potentially limiting the broader effectiveness of school-based interventions or policies.

Notwithstanding the small number of children who switched between school lunch and non-school lunch, the longitudinal findings corroborate findings from the cross-sectional analyses, suggesting that while school lunch shows benefits over non-school lunch in terms of school-time sugar and snacks consumption, the effects of switching from non-school to school lunch may not be universally beneficial in terms of diet quality. This suggests that further work is still required to improve the nutritional composition of school lunches, in particular to increase consumption of fruit and vegetables and decrease consumption of fried foods. This work should focus on helping schools implement the guidelines effectively. For example, although the school food standards include a standard on deep-fried food (no more than two deep-fried items per week), this was found to be met by only 35% of secondary schools in 2011, while only 23% and 3% of schools met standards for vegetables and fruit respectively (Nicholas, Wood, Harper, & Nelson, 2013).

## 5 Conclusions

Against a background of both positive and negative changes in overall diet from age 10 to age 14, our data show differential changes between diet consumed within school hours and outside of school hours, as well as differences in diet between those consuming school lunch and lunch obtained from elsewhere. School lunch consumption was associated with lower school-time sugar intake and savoury snack consumption, but also decreased fruit consumption and increased intake of fries. These findings suggest that any efforts to encourage improved school lunch take-up need to go alongside further work to improve school lunch provision to meet nutritional guidelines. Furthermore, the limited associations seen between school lunch consumption and total diet highlight the need for school-based policies to be implemented alongside wider changes to the whole food system.

## Ethics approval and consent to participate

Ethics approval for the SPEEDY study was obtained from the University of East Anglia Research Ethics Committee; written parental informed consent and written student assent were obtained before both measurement occasions.

## Funding

The SPEEDY study was funded by the National Prevention Research Initiative (http://www.npri.org.uk) (G0501294), consisting of the following funding partners: British Heart Foundation; Cancer Research UK; Department of Health; Diabetes UK; Economic and Social Research Council; Medical Research Council; Research and Development Office for the Northern Ireland Health and Social Services; Chief Scientist Office, Scottish Executive Health Department; Welsh Assembly Government; and World Cancer Research Fund. This work was also supported by the MRC (Unit Programme numbers MC_UU_12015/3; MC_UU_12015/4; MC_UU_12015/7; U105960389) and the Centre for Diet and Activity Research, a UK Clinical Research Collaboration (UKCRC) Public Health Research Centre of Excellence (RES-590-28-0002; MR/K023187/1). Funding from the British Heart Foundation, Cancer Research UK, Economic and Social Research Council, Medical Research Council, the National Institute for Health Research, and the Wellcome Trust, under the auspices of the UK Clinical Research Collaboration, is gratefully acknowledged. Diet diary data entry was partly funded by the Medical Research Council Human Nutrition Research Centre.

## Authors' contributions

EW, KC, MW and EvS designed the analyses, EW performed the analyses, and drafted the manuscript, all authors contributed to the interpretation of results and critically reviewed the manuscript. All authors read and approved the final manuscript.

## Competing interests

The authors declare that they have no competing interests.

## List of abbreviations

%E: percentage energy
ALSPAC: Avon Longitudinal Study of Parents and Children
BMI: Body mass index
EER: Estimated energy requirement
EI: Energy intake
g: grams
kJ: kilojoules
mg: milligrams
mle: maximum likelihood estimation
MJ: megajoules
SE: standard error
SPEEDY: Sport, Physical activity and Eating behaviour:Environmental Determinants in Young people
UK: United Kingdom
